# Advancing Toward a World Without Vision Loss From Diabetes: Insights From The Mary Tyler Moore Vision Initiative Symposium 2024 on Curing Vision Loss From Diabetes

**DOI:** 10.1167/tvst.14.5.12

**Published:** 2025-05-08

**Authors:** Ward Fickweiler, Konstantina Sampani, Dorene S. Markel, S. Robert Levine, Jennifer K. Sun, Thomas W. Gardner

**Affiliations:** 1 Beetham Eye Institute, Joslin Diabetes Center, Boston, MA, USA; 2 Department of Ophthalmology, Harvard Medical School, Boston, MA, USA; 3 Department of Medicine, Harvard Medical School, Boston, MA, USA; 4 Mary Tyler Moore Vision Initiative, Greenwich, CT, USA; 5 Department of Ophthalmology and Visual Sciences, Kellogg Eye Center, University of Michigan Medical School, Ann Arbor, MI, USA

**Keywords:** diabetic retinal disease, diabetic retinopathy, biomarkers

## Abstract

The Mary Tyler Moore Vision Initiative (MTM Vision) honors Mary Tyler Moore's commitment to ending vision loss from diabetes. Founded by Moore's husband, Dr. S. Robert Levine, MTM Vision aims to accelerate breakthroughs in diabetic retinal disease (DRD). At the MTM Vision Symposium 2024 on Curing Vision Loss from Diabetes, experts highlighted the urgent need for updated DRD staging systems, clinically relevant endpoints, and novel biomarkers to detect early disease changes. MTM Vision is advancing two clinical trials in collaboration with the DRCR Retina Network, launching a public awareness campaign, and welcoming Boehringer Ingelheim as the first founding industry member of its pre-competitive Consortium. Speakers emphasized big-data strategies and artificial intelligence (AI)-driven tools to improve DRD diagnosis, risk prediction, and personalized treatment. They also showcased new efforts to bridge academic discoveries with industry expertise, illustrating promising work on vascular regeneration and cellular senescence that may yield future therapies. The MTM Vision Biorepository and Resource Center is expanding tissue collections, enabling multi-omics analyses to study DRD mechanisms. Patient voices were central to the discussion, with calls for enhanced patient-reported outcomes, caregiver support, and broader education on DRD's risks. The symposium also underscored the importance of integrating mental health, quality of life measures, and ongoing patient input to guide clinical research.

## Introduction

The Mary Tyler Moore Vision Initiative (MTM Vision) was founded to honor the legacy of Mary Tyler Moore (1936–2017), an iconic actress, producer, and tireless advocate for diabetes awareness and research (see the Fig.). In partnership with her husband, Dr. S. Robert Levine, their efforts helped secure substantial funding for diabetes research with the ultimate goal to cure diabetes and its complications, including diabetic retinal disease. Following her passing, Dr. Levine established MTM Vision to help realize Mary's dream of a world without vision loss from diabetes. The mission of MTM Vision is to accelerate the development of new methods to preserve and restore vision in people with diabetes.^1^

**Figure.**
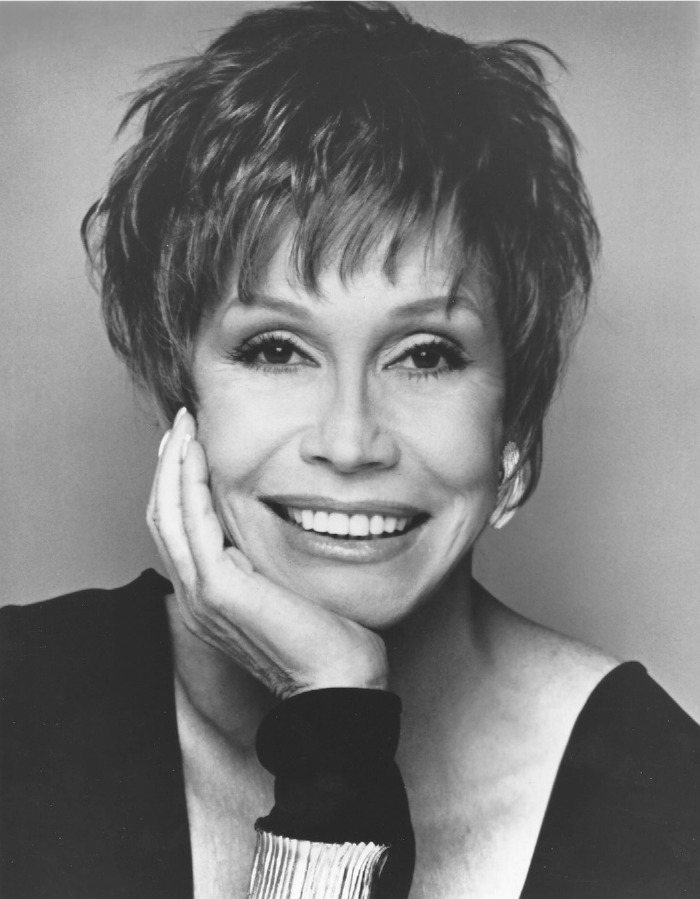
**Mary Tyler Moore.** Photographic rights owned by Dr. S Robert Levine, courtesy of Patrick Demarchelier.

Since its inception, MTM Vision has held multiple workshops and symposia to foster collaboration among experts and guide the strategic direction of the initiative from the identification of the most promising biomarkers for diabetic retinal disease (DRD)^1^^–^^7^ to best practices for building a large, shareable MTM Vision data lake.^8^ This report focuses on the Mary Tyler Moore Vision Initiative 2024 Fall Symposium, “Curing Vision Loss from Diabetes,” held on November 12, 2024, in Ypsilanti, Michigan, and hosted in partnership with the University of Michigan's Kellogg Eye Center and the Elizabeth Weiser Caswell Diabetes Institute, which set a collaborative stage for discussing advances and obstacles in DRD research, ultimately working toward MTM Vision's transformative goal of eliminating vision loss from DRD. The symposium attracted 184 attendees from around the world, with 139 people attending in person and 45 attending virtually. Sponsors of the Symposium were Verily, Regeneron, Bayer, Optos, ONL Therapeutics, LKC Technologies, Adaptive Sensory Technology, Biocryst, Boehringer Ingelheim, Kodiak, Anida Pharma, and Research to Prevent Blindness.

## The Need for Advancing Treatment and Prevention of DRD

The opening remarks of the Curing Vision Loss from Diabetes Symposium by Dr. Martin G. Myers, Jr., Director of the Caswell Diabetes Institute, highlighted the many achievements of the Mary Tyler Moore Vision Initiative to date, such as establishing a biorepository and launching critical research studies. Dr. Shahzad I. Mian, Chair of Ophthalmology and Visual Sciences at the Kellogg Eye Center, underscored MTM Vision as a collaborative approach in DRD research that can bridge discovery science, clinical trials, and public health policy to define DRD and set the stage for clinical success. Dr. S. Robert Levine, Founder and CEO of the Mary Tyler Moore Vision Initiative, emphasized the urgency of addressing DRD as a public health concern, citing recent research indicating that over 50% of individuals with youth-onset diabetes (both type 1 and type 2) show evidence of DRD by their mid-twenties.^9^ Dr. Levine detailed MTM Vision's progress over the past year, including two new clinical trials in collaboration with the DRCR Retina Network, several publications of MTM working group findings,^2^^–^^7^ organization of a data standardization workshop at ARVO 2024,^8^ and the launch of a public service announcement campaign to raise awareness about eye health in people with diabetes. A significant announcement was the introduction of Boehringer Ingelheim (BI) as the first founding member of the Mary Tyler Moore Vision Consortium. Marianne Laouri, the Global Asset Team Leader, Retinal Health of BI, highlighted the importance of this partnership that signifies a substantial step forward in uniting industry and academic research efforts to combat DRD. The opening remarks concluded with patient perspectives on DRD, which added a deeply personal layer to the MTM Vision mission, highlighting the profound impact of DRD on quality of life, including challenges with treatments and vision loss during pregnancy, and underscoring the critical need for advancements in treatment and prevention of DRD.

## Translating Science to Clinical Care for Diabetic Retinal Disease

To accelerate advances to preserve and restore vision in people with diabetes, an updated staging system and severity scale that takes into account the full spectrum of DRD and its impacts is needed. Recent advances in imaging and technology allow us to more fully characterize changes in the retina and to measure aspects of visual function beyond best corrected central visual acuity.

Jennifer Sun, MD, MPH, Science Co-Director of the Mary Tyler Moore Vision Initiative and Professor of Ophthalmology at Harvard Medical School, summarized efforts for an updated DRD staging system that would include retinal vascular function, neural function deterioration, biochemical markers, systemic health, and other parameters, including quality of life and visual function.^10^ The MTM Vision working group reviews have revealed significant gaps in current knowledge of endpoints in clinical care and research of DRD, particularly for guiding early-stage interventions.^2^^–^^7^ The first step in meeting the unmet need for new endpoints in DRD is to comprehensively validate potential endpoints of interest using state-of-the art imaging and functional testing in patients with diabetes, ranging from those who are newly diagnosed to those with proliferative disease or macular edema warranting treatment. As such, MTM Vision has developed 2 prospective, longitudinal clinical studies that will help validate potential endpoints of interest based on initial discussions at the MTM Vision Endpoints Workshop held in Ann Arbor in the Fall of 2022,^1^ and subsequent protocol development meetings and MTM Vision working group papers.^2^^,^^4^^–^^7^ The identification of priority variables (Table 1) provides a foundation for future studies aiming to validate these factors as reliable clinical endpoints. Over the last year, MTM Vision has refined these studies in collaboration with the DRCR Retina Network (DRCR), a National Institutes of Health-funded collaborative consortium of sites across North America, which performs clinical studies in retinal disease. Alongside MTM Vision, Breakthrough T1D (formerly JDRF) has committed support for these studies and for the data standardization efforts that will allow mapping of study variables to common data elements models.^8^ The first study will enroll a cohort of patients across the severity spectrum of DRD and characterize their functional and structural retinal changes over 4 years in the natural history of the disease. The second study will recruit patients who are beginning intravitreal anti-vascular endothelial growth factor (VEGF) therapy for center-involved diabetic macular edema and characterize baseline retinal abnormalities and longitudinal changes over a year of treatment. Study procedures are anticipated to include quantitative contrast sensitivity testing, RETeval electroretinography, objective field analyzer perimetry, ultrawide field fundus photography, fluorescein angiography, optical coherence tomography (OCT), and OCT angiography. Thus, the studies will define the relationships between standard structural parameters with changes in rod and cone photoreceptor pathways. The primary objective for these studies, which are both expected to start enrolling patients in 2025, is to define the ocular structural and functional characteristics of people with diabetes, covering a broad range of diabetes duration and disease severity in eyes over the natural history of the disease and in eyes undergoing treatment for diabetic macular edema. Additional discussions are underway to leverage other ongoing clinical research to provide supplemental endpoint validation datasets and to build an international network of centers able to draw on data from large cohorts to efficiently evaluate the prognostic and predictive benefit of risk factors, imaging technologies, functional assessments, and other novel endpoints in service of accelerating development and regulatory approval of potential new therapies for DRD. Building a strong partnership among academic, industry, and regulatory institutions is needed to encourage the incorporation of promising structural and functional variables, standardize variables across datasets in accordance with common data element models, promote sharing of clinical and research datasets, and support the development and validation of new primary endpoints for DRD.

**Table 1.** DRD Staging Update Priority Variables

	Ready (For Current Use or Within the Next 1–2 Y)	Promising (Unmet, But Defined Research Needs That Can be Accomplished Within Next 5 Y)	Potential (Unmet Research Needs That Will Need >5 Y to Accomplish)
Subclinical DRD (not clinically visible or evident)	Color fundus photographs (Std and UWF), Spectral domain optical coherence tomography with analytics	Optical coherence tomography angiography, Electroretinography (Full-field flash and flicker), Microperimetry, Objective field analyzer, Inflammation/Pro-I cytokines, Neuroprotection, Erythropoietin derivatives	Adaptive optics, RBP3, miR200, Mitochondrial/Mitophagy, slCAM1, sVCAM1, CRP
Early-stage Clinical DRD	Color fundus photographs (Std), Fluorescein Angiography (Std and UWF), Spectral domain optical coherence tomography with analytic s. Fenofibrate/lipid	Color fundus photographs (UWF), Optical coherence tomography angiography, Electroretinography (Full-field flash and flicker), Microperimetry, Contrast sensitivity, Low luminance visual acuity VE-PTP, TNFa, Inflammation/Pro-I Cytokines, Neuroprotection, erythropoietin derivatives	Adaptive optics, Objective field analyzer, RBP3, Soluble epoxide enolase, IL-6, IL-1b, slCAM1, sVCAM1, CRP, Erythropoietin derivatives, Mitochondrial/Mitophagy
Mid-stage Clinical DRD	Color fundus photographs (Std), Fluorescein Angiography (Std and UWF), Spectral domain optical coherence tomography with analytic s, Best corrected visual acuity, Fenofibrate/lipid, VEGF-A, Plasma kallikrein	Color fundus photographs (UWF), Optical coherence tomography angiography, Electroretinography (Full-field flash and flicker), Microperimetry, Contrast sensitivity, Low luminance visual acuity VEGF- R2, TNFa, Inflammation/Pro-I cytokines, Neuroprotection, Erythropoietin derivatives	Objective field analyzer, RBP3, DLL4, soluble epoxide enolase, IL-6, LRG1, EgIn1, L-1b, slCAM1, sVCAM1, CRP, Erythropoietin derivatives, Vascular Protection/Regeneration, Mitochondrial/Mitophagy, TNFa, CRP, sVCAM1
Late-stage Clinical DRD	Color fundus photographs (Std and UWF), Spectral domain optical coherence tomography with analytic s, Best corrected visual acuity, NEI VFQ-25 Ang2/Tie2, VEGF-A	Optical coherence tomography angiography, Full-field flash electroretinography, Microperimetry, Contrast sensitivity, Low luminance visual acuity, Static automated perimetry CCL2/MCP-1, CCR2/CCR5, VEGF- R2, IL-6, TNFa	RetCAT; RetDQo L; EQ-SD/VAS; DR-U Norrin, Notch-3, LRG1, EgIn1, CRP, IL-1b, slCAM1, sVCAM1, Vascular Protection/Regeneration, Mitochondrial/Mitophagy, TNFa, slCAM1, sVCAM1

Aude Couturier, MD, PhD, of Paris Cité University presented the EviRED project, a multicenter observational study conducted in France, which was initiated by Dr. Ramin Tadayoni.^11^ Using an artificial intelligence (AI)-assisted expert system, the EviRED project aims to replace current classification systems with individualized risk assessments for conditions, such as proliferative diabetic retinopathy (PDR) and diabetic macular edema (DME). EviRED’s model exemplifies how AI can potentially be used in the future for DRD care by improving diagnostic accuracy, personalizing follow-ups, and enabling earlier interventions.

Malvina Eydelman, MD, of the US Food and Drug Administration (FDA) discussed current endpoints for DRD which are mostly based on outdated guidelines from 2008, underscoring the need for updated, evidence-based criteria. She outlined the clinical validation and biomarker qualification pathways for broad regulatory and clinical application in DRD. Dr. Eydelman encouraged the research community to adopt a “backcasting” approach—working backward from desired future outcomes to identify the incremental steps needed today.

A panel consisting of Michael Abramoff, MD, PhD, University of Iowa, Jason McAnany, PhD, University of Illinois College of Medicine, and Sandy Puczynski, PhD, MTM Vision Lay Advisor, discussed the urgent need to establish new clinical endpoints and biomarkers for DRD. To achieve this, it is critical to: (1) bring together experts from various fields to agree on the most relevant and scientifically sound endpoints for DRD; (2) standardize data collection and sharing by utilizing common data elements and models to facilitate the comparison and validation of findings across studies and populations; (3) engage regulatory bodies early by collaborating with agencies like the FDA to ensure that new endpoints meet regulatory requirements and can be integrated into clinical trial designs; (4) incorporate patient perspectives including patient-reported outcomes to ensure that treatments address the symptoms and challenges most significant to those affected by DRD; and (5) leverage technological advancements utilizing AI and advanced imaging and visual function assessment techniques to enhance the detection, classification, and monitoring of DRD. Key priorities include consensus building by convening workshops to establish global agreement on clinical endpoints and biomarker standards, data sharing by promoting the interoperability of datasets across studies to enhance collaboration and reproducibility, and functional measures to expand research into early-stage neural dysfunction and its impact on daily activities. Thus, collaborative efforts and standardized methodologies will be key to translate scientific advancements into meaningful clinical outcomes that improve the lives of patients with diabetes and at risk of vision loss.

## Therapeutics Target Identification Through Human Tissue and Fluid ‘Omics

With support from the University of Michigan Central Biorepository, the MTM Vision-Biorepository and Resource Center (BRC) is collecting and storing ocular biospecimens by leveraging existing relationships with multiple national eye banks to procure ocular tissue samples. MTM Vision BRC is committed to provide essential resources, samples, and associated data to researchers worldwide to accelerate understanding of DRD. Under the leadership of Patrice Fort, PhD, Director of the MTM Vision BRC, the MTM Vision BRC is actively recovering, phenotyping, and characterizing ocular specimens. The center has collaborated with 9 eye banks across 23 states, successfully recovering 50 pairs of ocular samples including 12 non-diabetic and 38 diabetic eyes with 33% with no DRD, 43% with mild DRD, and 24% with severe DRD. A significant advancement is the integration of spatial analysis to evaluate perfusion and non-perfusion areas within retinal tissues. In collaboration with the Penn Institute for Biomedical Informatics, the MTM Vision BRC is developing a web-based platform to make these samples and associated data accessible to the research community. Users will be able to filter donor information based on factors like diabetes type, age, and gender, and access detailed clinical histories and imaging data. The MTM Vision BRC aims to foster collaboration and accelerate research by providing comprehensive datasets linked to various ‘omics analyses.

Roger Cone, PhD, Director of the Life Sciences Institute at the University of Michigan, highlighted the Michigan Drug Discovery as a model for bridging academic discoveries and clinical translation. The Michigan Drug Discovery offers a suite of tools and expertise to support researchers in target identification and validation of potential therapeutics. The program provides pilot funding enabling investigators to conduct high-throughput screening and initial hit-to-lead optimization. Industry professionals offer program management to ensure projects are strategically designed with appropriate endpoints and assays. Key facilities include the Center for Chemical Genomics, which offers high-throughput small molecule and genetic screening, and the Center for Structural Biology, providing resources for protein production and structural analysis. The natural products discovery program and cryo-electron microscopy facilities further enhance the capacity for detailed molecular investigations. These resources have already contributed to numerous FDA-approved drugs and clinical trial candidates originating from academic research.

Mike Sapieha, PhD, founder and Chief Scientific Officer SemaThea Inc., presented two examples of how basic research can lead to clinical advancements in treating retinal diseases. The first focused on revascularizing the retina by targeting Semaphorin 3A. By developing traps that sequester Semaphorin 3A, researchers were able to promote vascular regeneration and reduce pathological angiogenesis in animal models.^12^ To validate this approach, human ocular tissues and fluids from biorepositories were analyzed, confirming elevated levels of Semaphorin 3A in patients with PDR and DME.^13^^,^^14^ The technology was subsequently licensed to a pharmaceutical company, with clinical trials anticipated to commence in 2026. The second example addressed the role of cellular senescence in endothelial cells potentially contributing to retinal inflammation and vascular leakage.^15^^,^^16^ By identifying Bcl-xL, researchers developed a small-molecule inhibitor that selectively induces apoptosis in senescent endothelial cells.^17^ In diabetic mouse models, this treatment suppressed inflammation and restored vascular barrier function. Preclinical and phase 1 trial results suggest that Bcl-xL inhibition may have potential for patients with DME.^18^

A panel consisting of Lloyd Paul Aiello, MD, PhD, from Harvard Medical School, Eric Ng, PhD, from Eyebiotech Limited (a subsidiary of Merck), Subramaniam Pennathur, MD, from the University of Michigan, and Judy Hunt, MBA, from MTM Vision's Lay Advisory, highlighted the importance of integrating multi-tissue analyses and the differential effect of biochemical mechanisms between tissues. Challenges in tissue collection and imaging were also addressed, particularly the need to obtain comprehensive ocular imaging before tissue collection. Strategies to involve patients and families in donating tissues for research were discussed, recognizing the delicate balance between encouraging participation and managing expectations regarding tissue utilization. Transparent communication with the patient and family about the purpose and impact of tissue donations can build trust and participation. The gap between academia and industry in drug development was also a focal point of discussion. Bridging this gap requires collaborative efforts, leveraging the strengths of both sectors to accelerate the translation of research findings into viable therapeutics. Mitochondrial studies and connectomics were identified as promising areas for future exploration in DRD research.

## Big-Data and Artificial Intelligence Approaches to DRD

Founder and CEO of MTM Vision, S. Robert Levine, MD, underscored the importance of building reliable, diverse, and large-scale datasets that will enhance the MTM Vision roadmap to achieve the development of an updated, multidimensional DRD staging system (Table 2), which diagnoses DRD earlier, better quantifies DRD severity and risk of progression, and incorporates the patient perspective, visual function, retinal physiology, molecular milieu, and systemic factors.^10^ Furthermore, with the use of AI, MTM Vision aims to create a valid multimodal “DRD score” to predict progression and measure response to therapy, at all stages of disease. A fundamental step in this process is to establish a “data lake” to aggregate clinical study data, as well as retinal images functional data, and patient reported outcomes that can serve as a repository for additional data contributed by collaborators or consortium members, and include tools for data standardization, integration, harmonization, and analysis with the ability of cloud-based research analytics and training.^8^^,^^19^

**Table 2.** MTM Vision Initiative Roadmap

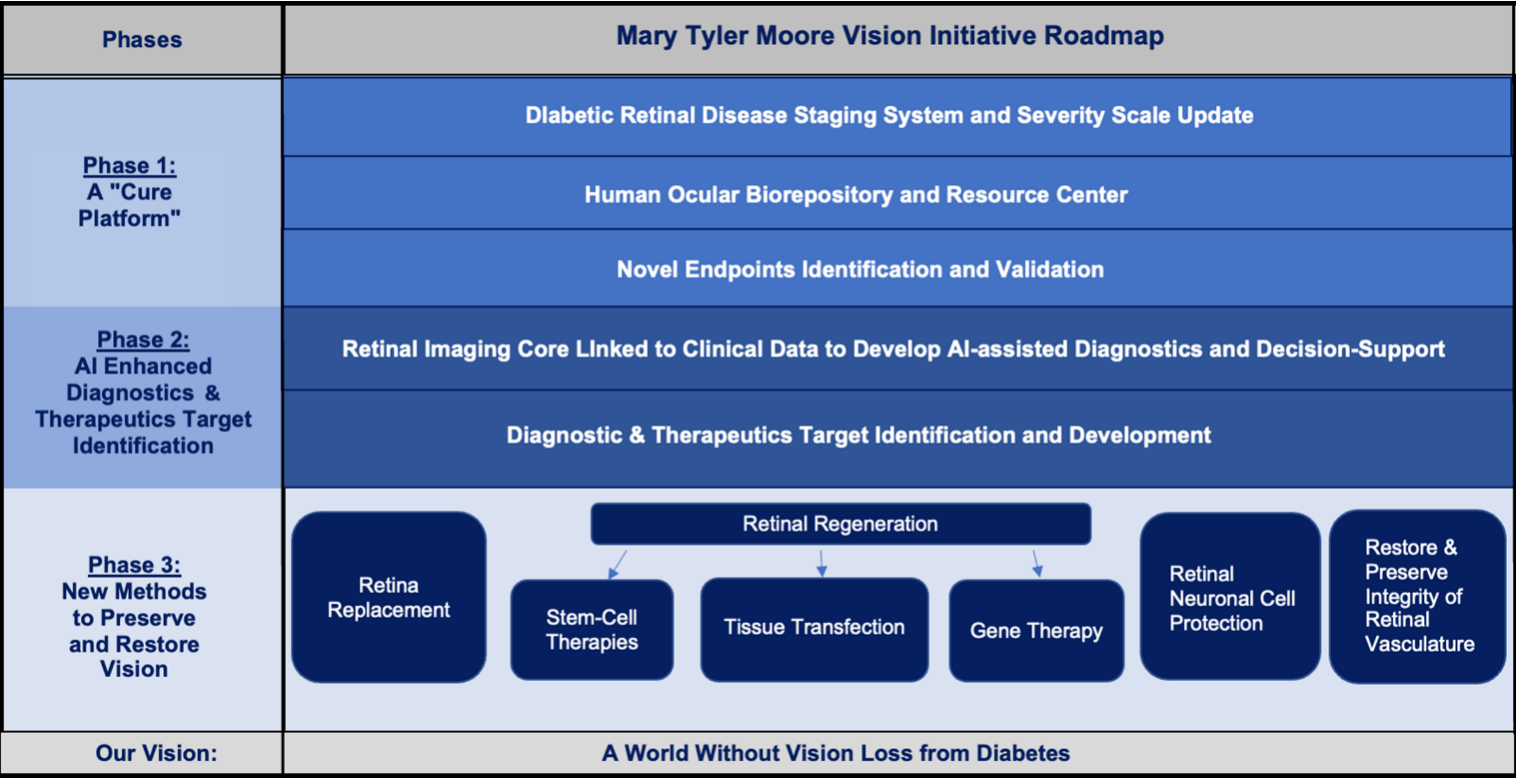

Tien-en Tan, MBBS (Hons), Mmed (Ophth), FRCOphth, FAMS, from Singapore National Eye Centre, Singapore Eye Research Institute, Duke-NUS Medical School, pointed out that ophthalmology was a very early adopter of AI and deep learning with development of algorithms for automatic screening for DRD on retinal color fundus photographs and leading to a number of commercially approved automated AI screening systems for diabetic retinopathy.^20^^–^^23^ Moving forward, there is an unmet need that AI should better address the individual DRD variability and better stratify DRD severity, risk of progression even in the early stages, and response to treatment. Furthermore, he highlighted the burden of DME treatment, the leading cause of vision loss from DRD,^22^^,^^24^ and emphasized the importance of ongoing collaboration between Singapore Eye Research Institute, the Institute for High Performance Computing, and DRCR Retina Network to train AI models using retinal imaging to predict “strong”^25^ treatment response in eyes with DME and vision impairment.

Brian VanderBeek, MD, MPH, MSCE, from the University of Pennsylvania/Scheie Eye Institute, is the inaugural recipient of the Research to Prevent Blindness – Mary Tyler Moore Vision Initiative Physician Scientist Award. He presented an overview on the major epidemiologic studies of DRD in the United States emphasizing the current gap in the trends over the past 20 years in the prevalence and incidence rates of DRD and DME.^20^^,^^24^^,^^26^^,^^27^ Research from his group demonstrated that DRD prevalence (through 2007) and incidence (through 2014) rates initially decreased but the rate of each has since doubled through 2022 due to the increased pool of patients with diabetes, increasing rates of the health insured population,^28^ and increased emphasis on DRD screening examinations. Notably, the prevalence rates of vision threatening retinopathy (VTDR), DME, and PDR increased 25% to 60% from 2007 to 2015 and decreased 10% since 2015 and the incidence rates decreased 45% to 69% over the past 20 years. Important factors to take under consideration were that the timing of decrease in approximately 2015 corresponds to increase in use of glucagon-like peptide-1 (GLP-1) agonists and the broadening use of anti-VEGF agents.

A panel consisting of Kerry Goetz, PhD, from the National Eye Institute, Tunde Peto, MD, PhD, from Queens University Belfast, and Chris German, PhD, an MTM Vision Lay Advisor, stressed the value of accessing large datasets and identifying high-quality data that we can use for training the AI models to effectively guide MDs on clinical diagnosis and management of DRD. Challenges on “real-world” data as contributor to clinical research data remain significant, but a large data size helps to mitigate errors. Aligning standards and ensuring data is accessible, interoperable, and consistently curated is crucial for integrating “real-world” data with clinical studies. Moreover, imaging data often is not linked to electronic health records, underscoring the importance of improving prospective data collections. Predictive models may be enhanced if additional clinical data can be integrated with current datasets to improve outcome prediction in DRD. However, there are no clear guidelines for translating AI algorithms into clinical use in ophthalmology, which prevents their implementation and acceptance by both clinicians and patients. Future efforts should focus on better integration of interpretable AI into the healthcare system.

## Awareness Crisis in DRD

Paolo Silva, MD, from Joslin Diabetes Center and Associate Professor of Ophthalmology at Harvard Medical School, discussed the need for improved adherence to recommended eye care guidelines among adults with diabetes to prevent and manage DRD effectively.^29^^,^^30^ Retinopathy unawareness and poor patient education about diabetes complications can lead to non-adherence with timely follow-up, increasing the risk of vision loss.^31^^,^^32^ He presented results from a study to assess self-reported patient awareness of DRD and concordance of scheduled follow-up with national guidelines in patients with diabetes over an extended period of time at a tertiary care diabetes center retinal telemedicine imaging program. The results showed that substantial discrepancies exist between DRD presence, patient awareness, and adherence of follow-up across all DRD severity levels and across all eye care provider types. Modification of medical care and education models may be necessary to enhance retention of ophthalmic knowledge in patients with diabetes and assure accurate communication between all healthcare providers.

## Gait Assessment in the Evaluation of Functional Vision

Amanda Bicket, MD, MSE, Assistant Professor at the University of Michigan, summarized gait features highlighting its complexity, the different quality metrics including pace, symmetry/smoothness, and variability, and its association with age, systemic health, and health-related quality of life. Gait can be studied by using floor mats to map footfalls or wearable sensors (inertial measurement units [IMUs]) to capture three-dimensional mobility. In eye disease, gait has been associated with glaucoma where patients with more visual field (VF) damage and contrast sensitivity (CS) loss demonstrate gait degradation in extreme or changing lighting.^33^ In DRD, gait changes can be an early indicator and potentially detect subclinical DRD. Thus, a planned pilot study, at Kellogg Eye Center at University of Michigan, will assess DRD-associated VF and CS loss on gait metrics and link gait quality with patient-reported outcomes, in patients with and without diabetic peripheral neuropathy, using IMUs in varied lighting conditions. Understanding gait changes may help provide necessary support to maintain patients’ independence and quality of life and potentially detect DRD and other ophthalmic diseases at an earlier stage.

## Developing a Patient-Reported Outcome for DRD

Fernanda Abalem, MD, MSC, PhD, from the University of Sao Paulo, Brazil, discussed the emotional distress due to diabetes and the unmet need for sensitive and effective novel endpoints that assess the efficacy of therapies for DRD that incorporate patient-reported outcome (PRO) measures.^3^^,^^34^ Although existing PRO measures are useful, gaps remain in addressing patients with DRD.^7^ Valuable insights of effective PROs were provided for inherited retinal degeneration with the development of Michigan Retinal Degeneration Questionnaire (MRDQ) and the Michigan Vision-related Anxiety questionnaire (MVAQ).^35^^,^^36^ In DRD, new PROs should be multi-dimensional tools assessing the disease impact and balancing between patient perspective and regulatory compliance. The development of an FDA compliant PRO involves 6 phases: focus group panels (phase 1); in-depth patient interviews (phase 2); content item generation that develops questions based on qualitative metrics (phase 3); cognitive interviews (phase 4); PRO administration (phase 5); and psychometric analysis and item reduction (phase 6).^37^^,^^38^

## Vitreous Analysis for Clinical Assessment, Staging, and Patient Selection for Clinical Trials

Jeffrey M. Sundstrom, MD, PhD, from Penn State University, highlighted the potential of using vitreous humor as a biofluid/tissue for evaluation of retinal diseases given vitreous’ proximity to the retina and its analogy to cerebrospinal fluid. The aim is precision medicine, enabling researchers to better select clinical trials participants and clinicians to identify which patients will be more likely to respond to specific treatments, thus reducing treatment burden and costs while improving outcomes. Dr. Sundstrom's group has developed a vitreous biopsy device able to acquire vitreous fluid in a clinical setting and perform quick proteomic vitreous analysis on-site, enabling optimization of individualized treatment.^39^^,^^40^ Ongoing efforts include preclinical testing of the biopsy device and studies for FDA approval. Mass spectrometry and Olink proteomic methods have expanded the understanding of the vitreous proteome, and identifying key proteins and pathways involved in DRD. The goals of these efforts are to develop a biomarker panel for high-risk non-proliferative diabetic retinopathy (NPDR), PDR, and angiogenesis, and to better predict treatment response.

## Future Directions: Restoring Vision in DRD: Lessons From the Inherited Retinal Diseases Field

Mark Pennesi, MD, PhD, Professor of Ophthalmology at Oregon Health & Science University and Director of Ophthalmic Genetics at the Retina Foundation, underscored the differences between inherited retinal diseases (IRDs) and DRD, particularly in the context of patient care and functional testing. Vision is multidimensional. Beyond measures of visual acuity, VF, CS, dark adaptation, and color vision, there are functional vision tests like reading speed and navigating ability, which are crucial for patient's self-efficacy and satisfaction. Many functional tests are used for IRDs, but getting a drug approved requires meeting strict regulatory definitions of clinically meaningful outcomes. Most IRD trials, especially gene therapy trials, have failed to meet these endpoints. New endpoints are being explored, such as low-luminance visual acuity, CS, tests examining rod and cone functions like optoretinography, mobility testing and hyperacuity tests on handheld devices like iPhones.^41^^,^^42^ PROs may further elucidate the clinical meaningfulness of these functional tests.

Rebecca Pfeiffer, PhD, from the University of Utah, introduced the term of pathoconnectomics in the field of IRDs, specifically in retinitis pigmentosa, that refers to the changes in wiring patterns of the outer and inner retina during and as a consequence of photoreceptor loss. Understanding details about the retinal rewiring process and the changes on neural retina prior to complete rod loss is crucial for advancing knowledge of retinitis pigmentosa as well as impacting the effectiveness of therapeutic intervention strategies.

Rachel Huckfeldt, MD, PhD, Assistant Professor of Ophthalmology at Harvard Medical School, presented on behalf of the Foundation Fighting Blindness Clinical Consortium Investigator Group and REDI Working Group. She gave an overview of the consortium highlighting its multicenter design modeled on the DRCR Retina network with the main goal to accelerate development of treatments for IRDs. The first natural history study, RUSH2A, has been completed and the 4-year results showed that the rate of change of mean sensitivity on static perimetry was a sensitive perimetric continuous measure, with functional transition points testing and microperimetry showing the largest within-eye changes, making them particularly useful for future IRD clinical trials design.^43^ Future directions include the investigation of additional novel sensitive endpoints, and the RUSH2A extension study of 7- and 9-year follow-up data analysis.

A panel consisting of James Weiland, PhD, from the University of Michigan, and Adriana Plevniak, MTM Vision Lay Advisor, underscored the importance of natural history studies in DRD and IRD as well as how crucial they are for identifying early endpoints and understanding disease progression. Functional testing has been found to be associated with IRD changes and may supplement the disease management but require high specificity. Patient-reported functional vision changes like adapting to light difficulties or contrast sensitivity loss are important to understand patient symptoms and real-life challenges essential for developing relevant tests.

## MTM Vision Ambassadors: Will and Kristen Flanary (“Dr. and Lady Glaucomflecken,” glaucomflecken.com)

The personal stories shared by the first MTM Vision Ambassadors, Will Flanary, MD, and Kristin Flanary, MA, served as a reminder that innovation in DRD care extends beyond technology and treatments but also includes communication, empathy, advocacy, and education as a precursor for change. Their personal stories illustrated systemic issues in healthcare, the power of storytelling, and the importance of engaging both patients and their families in clinical care.

Kristin Flanary introduced the concept of co-survivorship, highlighting the emotional, logistical, and psychological burdens experienced by family members of patients. This framework recognizes that the impact of diseases, such as DRD, extends beyond the individual to their support network, fundamentally altering lives. Asking a caregiver how they are doing can have profound impacts. Recognizing the broader context of patient care to include co-survivors can lead to more effective treatment plans and improved patient outcomes.

Will Flanary's online persona, Dr. Glaucomflecken, blends comedy and education to distill complex medical concepts into accessible, engaging content for a broad audience. Through his social media platforms, he highlights issues such as surprise billing, prior authorizations, and the administrative burdens that hinder patient care. His emphasis on education as a precursor to change resonated with the audience, illustrating how raising awareness of systemic problems and education can empower people to make their own informed decisions. Moreover, the power of his creative communication methods exemplified how public education about the importance of DRD on patients and caregivers’ lives can promote awareness and advocate for innovative research and systemic changes in DRD healthcare delivery.

## MTM Vision Consortium

The meeting also introduced the MTM Vision Consortium, developed as a collaboration between the University of Michigan and MTM Vision to engage industry in pre-competitive space research partnerships to advance research and enhance patient outcomes for DRD. Boehringer Ingelheim was announced as the first member of the MTM Vision Consortium. Shelby Unsworth, PhD, Michigan Medicine, underscored the MTM Vision Consortium’s mission: to combat diabetes-related vision loss through collaboration. This model fosters partnerships among academic institutions, industry stakeholders, and researchers to accelerate discoveries in prevention, diagnosis, and treatment of DRD. Marianne Laouri of Boehringer Ingelheim, the Consortium's founding industry partner, described how the company's commitment to advancing DRD research aligns with the Consortium's objectives, encouraging other industry and government entities to join the effort. The MTM Vision Consortium aims to create a platform for academic, industry, and nonprofit institutions to accelerate progress in addressing critical healthcare challenges for patients with DRD. By uniting diverse expertise and resources in the pre-competitive space, the initiative promises to drive transformative changes in the prevention and treatment of DRD.

## MTM Vision Communications, Collaborations, and Fund Development

A panel consisting of Betsy Cote from Joslin Diabetes Center, Chris Shoemaker from the University of Michigan, Maurine Slutzky from Stand-up to Cancer, and Sandy Puczynski, PhD, an MTM Vision Lay Advisor highlighted the importance of fundraising and philanthropy in health research and care. The first part focused on the philanthropy of successful organizations, like Michigan Medicine and Joslin Diabates Center, and underscored the significance of ethical engagement, awareness-building, and forming lasting relationships to drive successful fundraising and support for both research and patient care initiatives. Maurine Slutzky shared valuable insights of the organization's evolution and strategies emphasizing the key role of collaboration, strategic partnerships, and adaptability in successfully raising awareness and funds for cancer research. Eric Carlson, an award-winning documentary and film producer, summarized MTM Vision Initiative's achievements thus far. The first public service announcement in August 2024 reached over 100 million people and generated significant social media engagement. Future plans include partnerships with media entities for various events and content development, including re-creation of a classic episode(s) of the Mary Tyler Moore Show (with CBS) for broadcast for awareness-building and fund development, as well as development of a Broadway musical based on Mary Tyler Moore's life.
